# 

**Published:** 1894-10

**Authors:** 


### CONTENTS.

PROCEEDINGS.

Southern Dental Association—1894 .... 469
Every Man to his Trade .... 488
The American Dental Association .... 489
National Association of Dental Faculties .... 506

SELECTIONS.

Why are we Weary? .... 513
The Value of Sugar and the Effect of Smoking on Muscular Work.. 515
Chloroform Anæsthesia without Danger .... 516
The Healthiest Capital in the World .... 518
The Title of Professor .... 518

NOTICES.

New England Dental Society .... 519

EDITORIAL.

Tri-State Dental Meeting .... 520

